# A Disgusted Philanthropist

**Published:** 1885-10

**Authors:** 


### A DISGUSTED PHILANTHROPIST.

Our bilious neighbor of the *Columbus Medical Journal*, being unable to reply to the well merited excoriation this journal felt obliged to administer recently, on account of a palpable perversion of meaning, and misquotation of certain of our language, gets mad, and calls us "stupid," and says a good many hard things about us. To be sure they are not true, but we will let them pass, with the remark that to judge by the tone of all the *leading* journals towards us, he is entitled to priority in the discovery of our "stupidity." It seems that some persons, when they get mad, mistake abuse for argument.

It must have been while the mad fit was on him and he was reckless, that he "let the cat out of the bag," and furnished us a clue to his prejudices and bitterness against Texas—for, ever since the overthrow of the "fat" at New Orleans, to use his own elegant language—this writer has been spewing out bile against Texans *ad nauseam*, beginning with the application of vulgar epithets, to the Texas mothers, such as the New York *Record* felt obliged to omit, in quoting part of his article. He informs us in his August number, that "many years ago he ("ourself") and others went as voluntary missionaries to benighted Texas to enlighten the people,'' and that he would *now* like to enlighten *us*, were it not that " even the Gods [and the philanthropist (?) from Porkumbus?] are powerless against (the) stupidity'' of Texans—past and present.

(By the bye, our friend *denies* that he plays ''me too'' to the *New York Record*, and boasts that he actually ''had a tilt with the *Record*,'' on one occasion. No doubt he then ascertained he was likewise ''powerless'' against the *reverse* of stupidity. We may state parenthetically, that the traditional bull once ''had a tilt,'' with the locomotive also.)

There *was* a class of religious fanatics, who flocked to the South, just before the war, on some such absurd pretext as enlightening the poor Southerners, and while the ''irrepressible conflict'' was pending. They were not well received by the whites. Being looked upon as abolition emisaries, they were run out of the country, on suspicion of being *too intimate with the negroes*. Could it have been on this occasion that our benevolently inclined friend acquired his superior knowledge of ''kicks''?

